# Identification of two cancer stem cell-like populations in triple-negative breast cancer xenografts

**DOI:** 10.1242/dmm.049538

**Published:** 2022-06-23

**Authors:** Jun Nakayama, Hiroko Matsunaga, Koji Arikawa, Takuya Yoda, Masahito Hosokawa, Haruko Takeyama, Yusuke Yamamoto, Kentaro Semba

**Affiliations:** 1 Laboratory of Integrative Oncology, National Cancer Center Research Institute, Tokyo 104-0045, Japan; 2 Department of Life Science and Medical Bioscience, School of Advanced Science and Engineering, Waseda University, Tokyo 162-8480, Japan; 3 Computational Bio-Big Data Open Innovation Laboratory (CBBD-OIL), National Institute of Advanced Industrial Science and Technology (AIST), Tokyo 169-8555, Japan; 4 Research Organization for Nano & Life Innovation, Waseda University, Tokyo 169-8555, Japan; 5 Translational Research Center, Fukushima Medical University, Fukushima 960-1295, Japan

**Keywords:** Breast cancer, Cancer, Cancer stem cell, Spatial transcriptome, Xenograft model, scRNA-seq

## Abstract

Gene expression analysis at the single-cell level by next-generation sequencing has revealed the existence of clonal dissemination and microheterogeneity in cancer metastasis. The current spatial analysis technologies can elucidate the heterogeneity of cell–cell interactions *in situ*. To reveal the regional and expressional heterogeneity in primary tumors and metastases, we performed transcriptomic analysis of microtissues dissected from a triple-negative breast cancer (TNBC) cell line MDA-MB-231 xenograft model with our automated tissue microdissection punching technology. This multiple-microtissue transcriptome analysis revealed three cancer cell-type clusters in the primary tumor and axillary lymph node metastasis, two of which were cancer stem cell (CSC)-like clusters (CD44/MYC-high, HMGA1-high). Reanalysis of public single-cell RNA-sequencing datasets confirmed that the two CSC-like populations existed in TNBC xenograft models and in TNBC patients. The diversity of these multiple CSC-like populations could cause differential anticancer drug resistance, increasing the difficulty of curing this cancer.

## Abstract (precis)

**Summary:** We identified two types of cancer stem cell (CSC)-like populations in triple-negative breast cancer xenografts and patients. These CSC-like populations could potentially make tumors more drug resistant and thus more difficult to treat.

## INTRODUCTION

Breast cancer cells metastasize to multiple distant organs, such as the axillary lymph nodes, lungs, bone, liver and brain (Nakayama et al., 2021; Obenauf and Massagué, 2015). In particular, metastasis to axillary lymph nodes is an indicator of cancer grade in breast cancer patients (Giuliano et al., 2011). Most breast cancer tissues, including distant metastases, exhibit genetic heterogeneity (Gillies, 2022; McGranahan and Swanton, 2017). Single-cell analyses have revealed that cancer cells evolve through the acquisition of genomic mutations in the primary tumor and metastases (Echeverria et al., 2018; Yates et al., 2017). Most previous analyses have been performed using isolated cancer cells and stromal cells from cancer tissues. Thus, the cell–cell interactions between cancer cells and stromal cells remain to be analyzed. In recent studies, current single-cell analysis and spatial transcriptome technologies have revealed the heterogeneity of cell–cell interactions between cancer cells and stromal cells *in situ* (Andersson et al., 2021; Rao et al., 2021; Wu et al., 2021b); however, further analysis is needed to elucidate the nature of tumor heterogeneity.

Comprehensive gene expression analysis of metastases harvested from ∼500 specimens of various cancer types and metastatic organs (MET500 cohort) has suggested that metastatic tissues can be divided into several categories [e.g. proliferative or epithelial–mesenchymal transition (EMT)-like/inflammatory] (Robinson et al., 2017). In particular, some samples were found to show signatures of more than one category, suggesting that these samples have micro-intratumor heterogeneity. Heterogeneous tumors contain a small subpopulation of cancer stem cells (CSCs) able to induce anticancer drug resistance and metastasis (Oskarsson et al., 2014; Turdo et al., 2019; Weiss et al., 2022). To clarify such heterogeneity, microtissue sectioning using laser capture microdissection has often been performed (Civita et al., 2019). This method has several disadvantages, including the laborious and time-consuming nature of sample handling and a high risk of RNA degradation. Thus, in previous work, we developed a system involving automated tissue microdissection punching followed by transcriptomic analysis of the tumor microtissue (Yoda et al., 2017). This site-specific rapid sampling method by a hollow punching needle from frozen tissue enables low-cost molecular analyses to be performed on low-resolution spatially resolved tissue specimens. To analyze the expressional heterogeneity in microtissues from the primary tumor and axillary lymph node metastasis, we performed analysis of the spatial microtissue transcriptome in a xenograft model with the triple-negative breast cancer (TNBC) cell line MDA-MB-231. We focused on the expression profiles of known metastasis-promoting genes and CSC markers in dissected microtissues.

## RESULTS

### Sampling microtissues from primary tumor and axillary lymph node metastasis in MDA-MB-231 xenografts

**Fig. 1.**
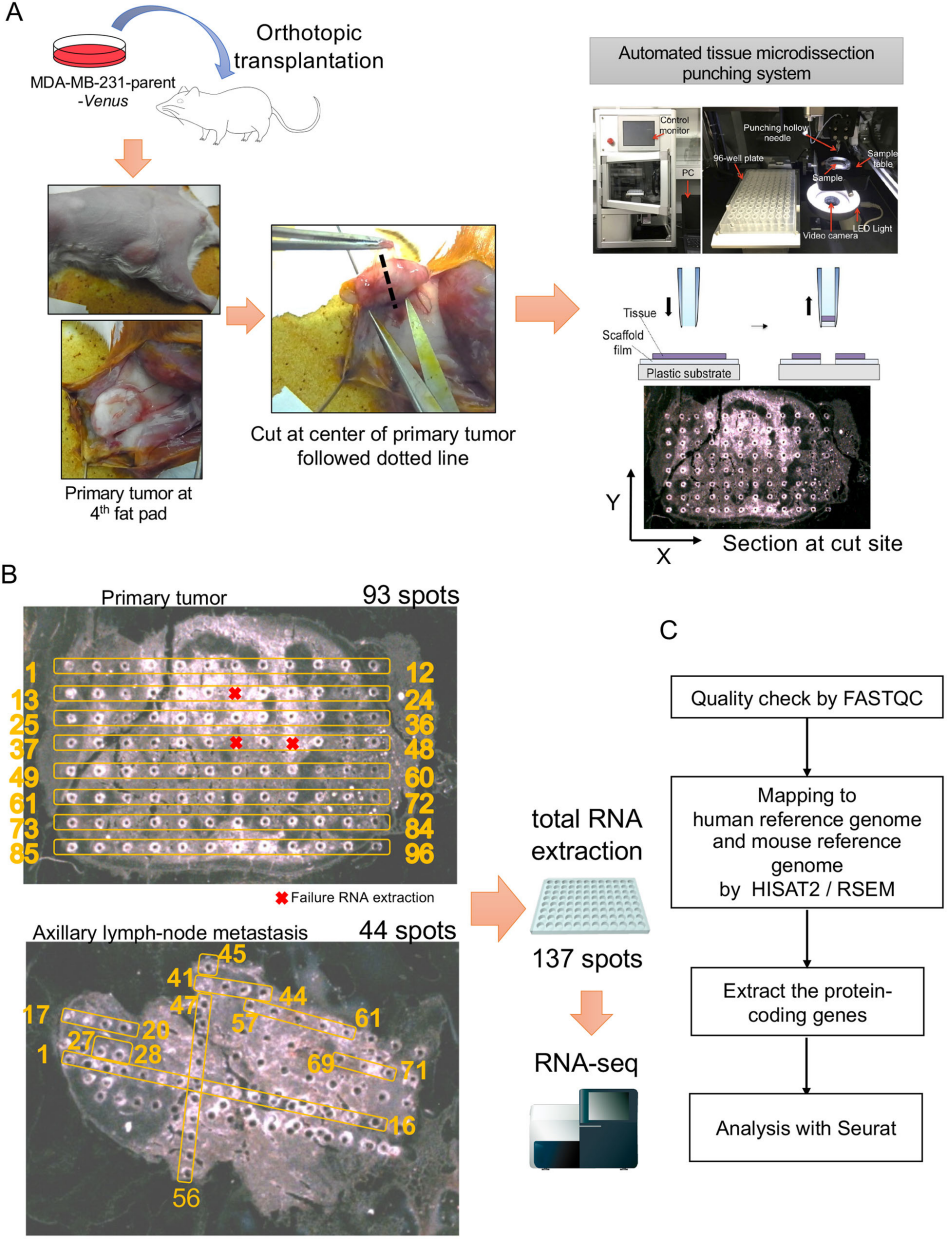
**Microtissue sectioning of the primary tumor and the axillary lymph node metastasis from the TNBC xenograft model.** (A) Experimental flowchart of the spatial transcriptomics analysis. The human triple-negative breast cancer (TNBC) cell line MDA-MB-231-parent-*Venus* was transplanted orthotopically into a female NOD-SCID mouse. After 8 weeks, the primary tumor was harvested, and the center of the tumor was sectioned. After 4 weeks, axially lymph node metastases were harvested and sectioned from the same mouse. Sectioning was performed by an automated tissue microdissection punching system with a 100 μm needle. (B) In total, 93 microspots were sectioned from the primary tumor, and 43 microspots were sectioned from axillary lymph node metastases. RNA was extracted from a total of 137 spots. (C) Flowchart of the transcriptome analysis. A quality check was performed by FASTQC. The reads were mapped to the human reference genome and the mouse reference genome by HISAT2 and RSEM. Protein-coding genes were selected for analysis with Seurat.

Primary tumors and axillary lymph node metastases were harvested from NOD-SCID mice with MDA-MB-231-parent-*Venus* cell line xenografts. We subjected the sliced tissues to microtissue dissection by an automated tissue microdissection punching system (Fig. 1A). RNA was successfully recovered from the microtissues collected at 93 spots in the primary lesion and 44 spots in axillary lymph node metastasis using a microtissue automatic sampling device (Fig. 1B). In samples of this size, although the number of cells present in the tumor tissue varies, it can be inferred that several to ∼10-30 cells are present in each spot (Yoda et al., 2017). RNA-sequencing (RNA-seq) analysis was performed on the total RNA extracted from each spot. We checked the quality of the FASTQ files by FASTQC. Total RNA samples contained RNA from human cancer cell lines and RNA from mouse stromal cells in the tumor microenvironment. Therefore, the obtained sequences were mapped to both the human reference genome and the mouse reference genome by HISAT2 (Kim et al., 2019). Protein-coding genes (human, 19,961 genes; mouse, 22,050 genes) were extracted as transcripts per million (TPM) for spatial transcriptome analysis with Seurat (Fig. 1C; Fig. S1A-C) (Butler et al., 2018; Stuart et al., 2019).

### Microtissue transcriptomics analysis reveals two types of CSC-like populations

**Fig. 2.**
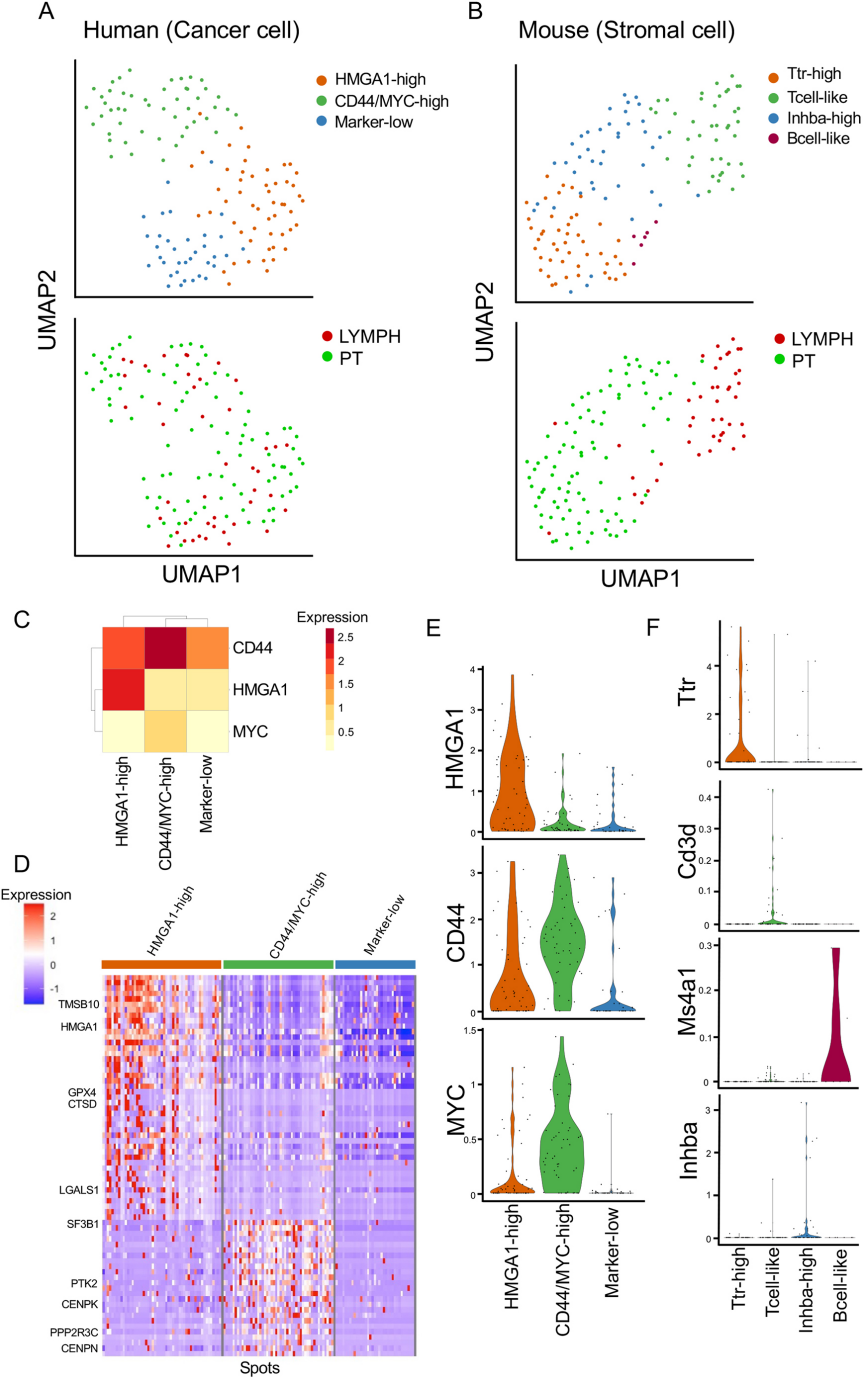
**Transcriptome profiling of the clusters in each microspot using cancer RNA and stromal RNA.** (A) Uniform manifold approximation and projection (UMAP) plot of 137 human (cancer cell) spots in the primary tumor and lymph node metastasis. (B) UMAP plot of 137 mouse (stromal cell) spots in the primary tumor and lymph node metastasis. (C) Heatmap of *CD44*, *HMGA1* and *MYC* expression in each human cell cluster. (D) Heatmap of differentially expressed genes (DEGs; adjusted *P*-value<0.05 and pct.1−pct.2>0.1) in each human cell cluster. (E) Violin plots of expression levels of cancer marker genes, *CD44*, *HMGA1* and *MYC*. (F) Violin plots of expression levels of stromal marker genes, *Ttr*, *Cd3d*, *Ms4a1* and *Inhba*.

The clustering analysis and uniform manifold approximation and projection (UMAP) plots showed three clusters of cancer cells (transcripts mapped to the human reference genome) and four clusters of stromal cells (transcripts mapped to the mouse reference genome) in the microspots dissected from primary tumors and axillary lymph node metastases (Fig. 2A,B; Table S1). Next, we evaluated the expression of CSC markers to focus on the CSC populations in the primary tumor and metastatic lesion (Oskarsson et al., 2014). We found that human cancer clusters showed specific gene expression patterns for high-mobility group AT-hook 1 (*HMGA1*) and *CD44* (Fig. 2C,E; Table S2). CD44 and HMGA1 are well-known markers of CSCs in breast cancer (Liu et al., 2010; Pegoraro et al., 2013). *CD44* was broadly expressed in all human cell clusters, whereas *HMGA1* was highly expressed only in HMGA1-high clusters (Fig. 2C). The upstream enrichment analysis using Metascape (Zhou et al., 2019) showed that the differentially expressed genes (DEGs) of the *CD44*-expressing clusters were enriched in MYC-targeted genes (Table S3). Consequently, these human cell clusters were named HMGA1-high, CD44/MYC-high and marker-low. The DEGs in the human cell clusters were extracted and visualized as a heatmap (Fig. 2D; Table S2). The heatmap showed that the HMGA1-high cluster expressed *TMSB10* (Zhang et al., 2017), *CTSD* (Ashraf et al., 2019) and *LGALS1* (Balestrieri et al., 2021; Jung et al., 2007), which are correlated with poor prognosis in breast cancer. The human CD44/MYC-high cluster expressed *CENPK* (Lee et al., 2015) and *CENPN* (Wu et al., 2021a), which regulate the cell cycle and cell division in cancer. The human marker-low clusters showed low expression levels of these genes. These results suggested that two types of CSC-like populations existed in the MDA-MB-231 xenografts.

To determine the biological function of DEGs, we performed upstream analysis, gene ontology (GO) enrichment analysis and pathway enrichment analysis using Metascape. We performed pathway and GO enrichment analyses focused on the two CSC-like clusters that had upregulated DEGs (Fig. S2, Table S4). Amide metabolites, VEGFA–VEGFR signaling and ribonucleoprotein complex biogenesis were enriched in both clusters. The DEGs in the CD44/MYC-high clusters were enriched in many terms related to the cell cycle, cell division and ribosomal biogenesis. In the HMGA1-high clusters, the terms ribosome and TRBP complex were significantly enriched.

**Fig. 3.**
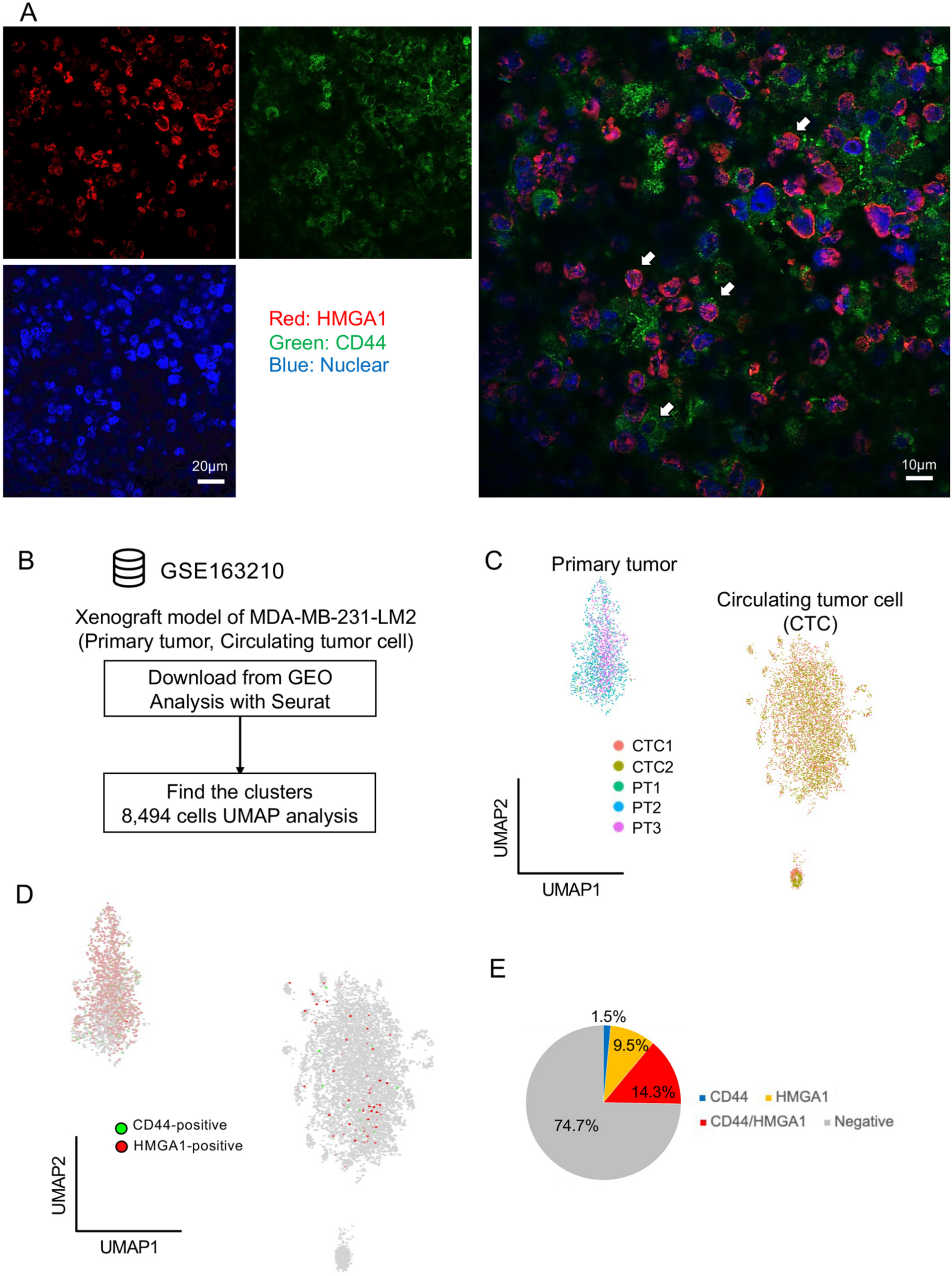
**Two CSC-like populations detected by immunostaining and scRNA-seq analysis.** (A) Representative images of CD44 and HMGA1 immunostaining in primary tumor sections from the MDA-MB-231 xenograft model. Red, HMGA1; green, CD44; blue, nuclei. Scale bars: 20 μm (left), 10 μm (right). White arrows represent cancer cells with independent expression or co-expression. (B) Flowchart of public single-cell RNA-sequencing (scRNA-seq) reanalysis using the GSE163210 dataset. (C) UMAP plot of MDA-MB-231-LM2 xenograft tumors and circulating tumor cells. (D) Expression of *CD44* and *HMGA1* in the UMAP plot. (E) Pie chart of the cancer stem cell (CSC)-like population proportion in MDA-MB-231-LM2 xenografts.

The expression of CD44 and HMGA1 in transplanted sections was confirmed with fluorescence immunostaining (Fig. 3A). CD44 and HMGA1 were detected in primary tumor sections. There were cells that expressed CD44 and HMGA1 independently and cells that co-expressed both CD44 and HMGA1. To confirm the existence of two CSC-like populations in the MDA-MB-231 xenograft model, we reanalyzed the public single-cell RNA-seq (scRNA-seq) samples of primary tumors and circulating tumor cells (CTCs) from the MDA-MB-231-LM2 xenograft model (Moravec et al., 2021 preprint). We downloaded the GSE163210 dataset from the Gene Expression Omnibus (GEO) database and reanalyzed it with Seurat/R (Fig. 3B). We extracted 8494 cancer cells from the dataset with UMAP visualization (Fig. 3C). The scRNA-seq analysis revealed that there were HMGA1-high, CD44-high and double-positive (HMGA1- and CD44-positive) populations in both the primary tumors and CTCs in the xenograft model (Fig. 3D,E). These results suggested that there were two types of CSC-like populations expressing CSC marker genes in the MDA-MB-231 xenograft model.

The mouse stromal clusters showed specific gene expression patterns for transthyretin (*Ttr*), *Cd3d* (T-cell marker), membrane-spanning 4-domains a1 (*Ms4a1*; B-cell marker) and inhibin subunit beta A (*Inhba*; a subunit of both activin and inhibin) (Fig. 2F; Fig. S3A). *Ttr* and *Inhba* were highly expressed in their respective specific clusters; therefore, these mouse clusters were named ‘Ttr-high’, ‘Tcell-like’, ‘Inhba-high’ and ‘Bcell-like’. Enrichment analysis of the DEGs of the mouse clusters showed that only the Tcell-like clusters had many enriched terms (Fig. S3B, Table S5). The citric acid [tricarboxylic acid (TCA)] cycle, chemical stress response and fatty acid oxidation were enriched in the Tcell-like populations.

### Microspot spatial and cell cycle analyses

**Fig. 4.**
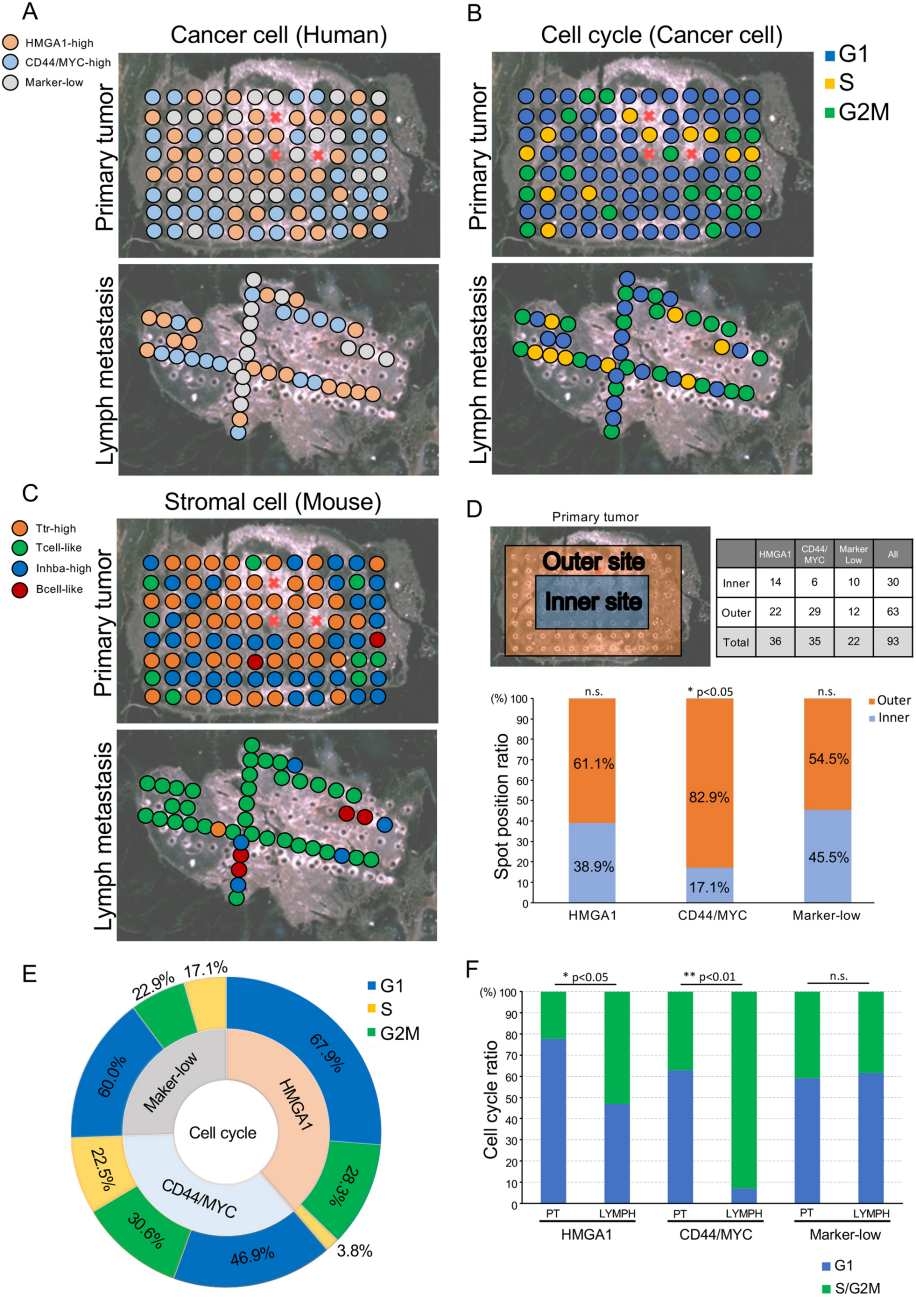
**Spatial analysis revealed the aggressive proliferation of CSC-like populations in the lymph node metastasis.** (A) Spatial transcriptomics analysis of human (cancer cell) clusters in the primary tumor and the lymph node metastasis. (B) Cell cycle phase of cancer cells in the primary tumor and lymph node metastases. (C) Spatial transcriptomics analysis of mouse (stromal cell) clusters in the primary tumor and lymph node metastasis. (D) Spatial analysis of human (cancer cell) clusters with the chi-squared test; *P<0.05; n.s., not significant. (E) Sunburst plot of the cell cycle in human cancer cell clusters. (F) Bar plot of cell cycle phases in the primary tumor and lymph node metastasis. Fisher's exact test; *P<0.05; **P<0.01; n.s., not significant.

Next, we performed spot analysis with the spatial information to determine the spatial heterogeneity in the xenografts (Fig. 4A-C). Interestingly, although three human cell clusters were present in both the primary tumors and the lymph node metastases (Fig. S4A), the mouse stromal clusters showed a site-specific pattern (Fig. S4B). Most of the Ttr-high clusters were observed in the primary tumors, whereas most of the Tcell-like clusters and Bcell-like clusters were found in the lymph node metastases. Human CD44/MYC-high cancer cells tended to localize the outside of the primary tumor (Fig. 4A,D).

Cell cycle analysis of the cancer cells showed that cell proliferation occurred outside of the primary tumor and at sparse sites among the lymph node metastases (Fig. 4B). Approximately 50% of the cells in the CD44/MYC-high clusters and 30% of the cells in the HMGA1-high clusters were actively undergoing cell division (Fig. 4E; Fig. S4C, Table S6). These results suggested that the cells that had originated from the two CSC-like clusters, leading to cancer expressional heterogeneity. Next, a comparative analysis of the cell cycle in the primary tumor and lymph node metastasis showed that the two CSC-like clusters (HMGA1-high and CD44/MYC-high) had an increased cell division index in lymph node metastasis (Fig. 4F). In contrast, the marker-low clusters did not have an altered cell cycle index in either location. These results suggested that the CD44/MYC and HMGA1 CSC-like populations in metastatic tissues proliferated aggressively.

Mouse stromal cell localization assessment showed that most of the Tcell-like clusters were present throughout the entire lymph node metastases, and the Tcell-like clusters also existed outside the primary tumor. Most of the Ttr-high clusters were sparsely present throughout the primary tumor (Fig. 4C). The cells in the mouse Tcell-like clusters and Inhba-high clusters showed an active cell cycle (Fig. S4D-F).

### Two CSC-like populations in TNBC patients

**Fig. 5.**
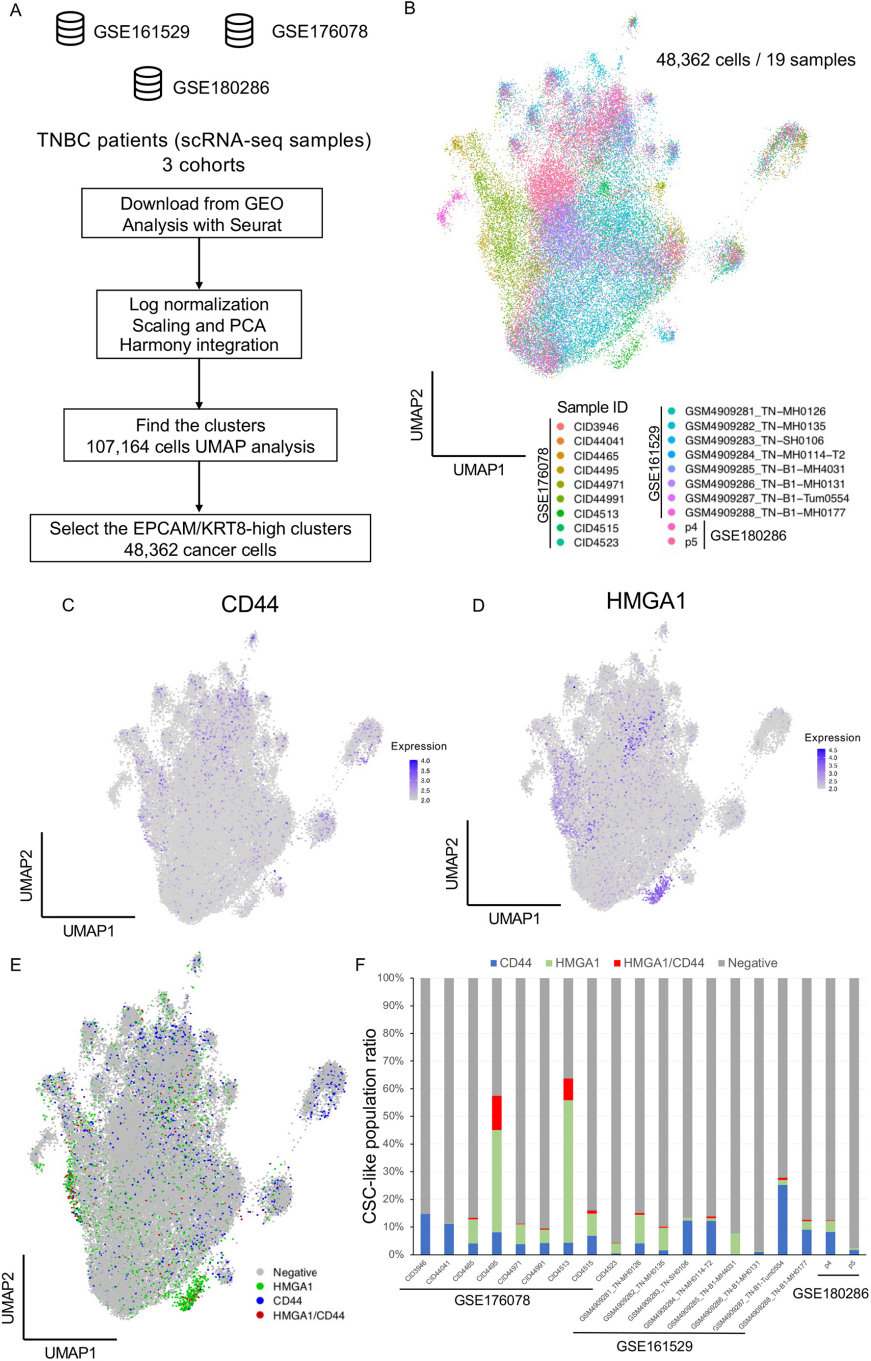
**Reanalysis of clinical scRNA-seq with CSC-like signatures.** (A) Flowchart representing the strategy of reanalysis using public scRNA-seq datasets. We downloaded GSE161529, GSE176078 and GSE180286 including scRNA-seq data from 19 TNBC patients. The integrated datasets were analyzed with Seurat. Log normalization, scaling, principal component analysis (PCA) and UMAP visualization were performed following the basic protocol in Seurat. To extract the cancer cells, cells expressing EPCAM/KRT8 (epithelial marker) were filtered. Cancer cells (48,362 cells) were extracted from 107,164 single cells. (B) UMAP plot of cancer cells from 19 TNBC patients with sample IDs. (C,D) Expression analysis of *CD44* (C; expression level>2) and *HMGA1* (D; expression level>2) with UMAP plots. (E) UMAP plot of CD44-high, HMGA1-high, HMGA1/CD44-high and marker-negative cancer cells. (F) The proportion of cancer cells that expressed *CD44* and *HMGA1* are represented in a bar plot.

To confirm our findings in clinical samples, we reanalyzed three integrated public scRNA-seq datasets (Pal et al., 2021; Wu et al., 2021b; Xu et al., 2021). We analyzed the integrated scRNA-seq dataset of 19 TNBC samples (Fig. 5A) and extracted 48,362 cancer cells from the dataset with UMAP visualization (Fig. 5B; Fig. S5A). Expression analysis detected HMGA1-high cancer cells (HMGA1 expression level>2), CD44-high cancer cells (CD44 expression level>2) and double-positive cancer cells in the integrated TNBC cohorts (Fig. 5C,D; Fig. S5B). Most of the TNBC patients had two CSC-like populations and a double-positive population (Fig. 5E,F; Table S7).

## DISCUSSION

Spatial transcriptomics technologies have enabled us to reveal the *in situ* expressional profiles and microheterogeneity of cancer (Rao et al., 2021). In particular, in a xenograft model, both human-derived RNA and mouse-derived RNA can be analyzed simultaneously and individually by mapping the sequence reads to a human genome reference or mouse genome reference (Bradford et al., 2013; Callari et al., 2018). In this study, by combining microtissue sampling and the isolation of human–mouse gene expression by mapping, we revealed the expressional heterogeneity of cancer cells and stromal cells in MDA-MB-231 primary tumors and axillary lymph node metastases. We observed two types of CSC-like populations in both the primary tumors and lymph node metastases. One of the CSC-like populations expressed *CD44* and *MYC*. The *CD44* gene is a well-known CSC marker in breast cancer (Liu et al., 2010; Marotta et al., 2011; Sheridan et al., 2006). The other CSC-like population, the HMGA1-high cluster, was observed in both the primary tumors and lymph node metastases. HMGA1 promotes tumor initiation, cancer stemness and metastasis in TNBC (Huang et al., 2015; Pegoraro et al., 2013; Shah et al., 2013). Enrichment analysis of DEGs showed upregulation of terms related to ribosome processes. The upregulation of translation and ribosomal processes may promote distant metastasis in breast cancer (Ebright et al., 2020). scRNA-seq analysis and immunostaining of MDA-MB-231 xenografts confirmed the two types of CSC-like populations. Spatial transcriptomics of mouse genes showed Inhba-high stromal populations in the xenograft model. Inhba is a member of the TGF-β superfamily (Bloise et al., 2019). INHBA is upregulated in breast tumors and induces EMT, tumor growth and distant metastasis (Bashir et al., 2015; Kalli et al., 2019). Most Inhba-high populations also existed in the primary tumor. Our results suggest that stromal expression of Inhba enhanced tumor growth in the MDA-MB-231 primary tumors. In addition, the punching microdissection system used in this study enables the extraction of only the parts of a section, which is useful for analysis at low cost. This system might be applied in analysis for extraction of fresh RNA samples instead of laser-capture microdissection methods.

Reanalysis of scRNA-seq of the CTCs in MDA-MB-231-LM2 xenografting mouse revealed that the number of CSC-like populations in CTCs was small; however, a large number of CSC-like populations was identified in the lymph node metastasis by our spatial transcriptomics. Our results showed that CSC-like populations significantly proliferated in the metastatic region, suggesting that CSC-like populations were increased by aggressive proliferation in the metastatic regions from a small number of disseminated tumor cells. In the integrated clinical scRNA-seq analysis, both types of CSC-like populations were observed in single-cell analysis of TNBC patients. Our results suggested that the co-existence of these multi-CSC-like populations makes curative treatment difficult and causes anticancer drug resistance in the clinic. Additionally, we extracted the DEGs from these CSC-like populations in TNBC patients, suggesting that some DEGs (*CD44*, *SRGN*, *CXCR4*, *TXNIP*, *ANXA1*, *HNRNPH1*, *DDX5* and *ITM2B* in the CD44/MYC-high cluster; *HMGA1*, *GAPDH* and *RPLP0* in the HMGA1-high cluster) of CSC-like populations were consistent between the clinical patients and our spatial transcriptomics (Table S8). Several samples from the clinical patients were enriched CSC-like populations. The difference in populations is caused by sampling bias in the GSE176078 dataset. It is considered that the patients' samples with a large number of CSC-like populations might have been sampled from an area enriched with CSC-like cancer cells. This is a methodological limitation of reanalysis using a public dataset.

Our study has limitations. Several spots did not contain enough RNA for analysis or exhibited bias toward either human RNA or mouse RNA (Fig. S1A). Thus, one limitation of this sampling method is that some spots have a biased cell type or no cells. Regarding the read counts (nCount_RNA) of each cluster, there were lower counts for the human marker-low cluster, mouse Ttr-high cluster, mouse Inhba-high cluster and mouse Bcell-like cluster than for other clusters (Fig. S1B,C). The limitations of cell type bias and low RNA extraction efficiency caused these low transcript counts. In particular, the marker-low human cluster and Ttr-high mouse cluster had the lowest nCount_RNA. This result suggests that these clusters may have included dead/dying cells. Tumor tissue has spatial heterogeneity, including in necroptotic/apoptotic areas, which is one of the limitations of spatial transcriptome analysis. We also detected different nCount_RNA and features, so it was difficult to define the exact characteristics and cell types in the tissue at single-cell resolution. This is a technical limitation of spatial transcriptomics. More high-resolution spatial transcriptomics analyses are needed to define the cluster characteristics at single-cell resolution, as has been recently developed (Srivatsan et al., 2021). For reproducibility of these spatial transcriptomics in MDA-MB-231 xenografts, we reanalyzed primary tumors and metastatic lesions from other mice as pilot study samples. The microspots including test trial samples showed three human clusters, which were classified into CD44/MYC, HMGA1 and marker-low clusters (Fig. S6).

In conclusion, our study showed that there are two types of CSC-like populations in MDA-MB-231 xenograft models and TNBC patients. The presence of these CSC-like populations could potentially make tumors more drug resistant and thus more difficult to treat. More effective therapies need to be developed through the elucidation of intratumor heterogeneity. In addition, our spatial transcriptomics methods will be helpful for the diagnosis, further identification of biomarkers and elucidation of the essential characteristics of cancer.

## MATERIALS AND METHODS

### Cell culture

The MDA-MB-231-parent-*Venus* cell line was cultured in RPMI-1640 medium (Fujifilm Wako, Osaka, Japan) supplemented with 10% heat-inactivated fetal bovine serum (FBS; Fujifilm Wako), 100 µg/ml streptomycin (Meiji Seika Pharma Co. Ltd., Tokyo, Japan) and 100 U/ml penicillin (Meiji Seika Pharma) at 37°C with 5% CO_2_.

### Animal studies

A breast cancer xenograft model was established in NOD.CB-17-*Prkdc^scid^*/J mice (NOD-SCID; Charles River Laboratories Japan, Inc., Kanagawa, Japan) by orthotopic transplantation as previously described (Nakayama et al., 2017). A total of 1.0×10^6^ cells were injected into the fourth fat pad of NOD-SCID mice. The primary tumor was removed 8 weeks after transplantation. An axillary lymph node metastasis was sampled 2 weeks after removing the primary tumor. The growth of the primary tumors and metastases was monitored by bioluminescence using an *in vivo* imaging system (IVIS-XRMS, PerkinElmer, Waltham, MA, USA). For bioluminescence monitoring by *in vivo* imaging system (IVIS), mice were anesthetized with 2.5% isoflurane (Fujifilm Wako) and intraperitoneally injected with 3 mg D-luciferin (Gold Biotechnology Inc., Olivette, MO, USA) in 200 µl PBS as previously described (Han et al., 2020; Kuroiwa et al., 2020). The harvested organs were placed in ice-cold PBS (Fujifilm Wako), embedded in Super Cryoembedding Medium (SECTION-LAB, Hiroshima, Japan) using liquid nitrogen and stored at −80°C until sectioning.

Animal experiments in this study were conducted under the approval of the Animal Committee of Waseda University (2017-A043a) and conformed to Animal Research: Reporting of *In Vivo* Experiments (ARRIVE) guidelines. Approved protocols were strictly adhered to..

### Microtissue dissection and RNA-seq analysis

Microtissue sampling was performed by an automated tissue microdissection punching system as previously described (Yoda et al., 2017). Frozen sections were sliced at a thickness of 20 μm and transferred to an LMD film II (SECTION-LAB). Microspots were sampled with a 100 μm needle in the dissection instrument. RNA-seq was performed by Illumina HiSeq as previously described (Yoda et al., 2017).

### Mapping and quality check

Transcriptome analysis was performed with HISAT2 version 2.0.5 (Kim et al., 2019) and RSEM version 1.3.0 (Li and Dewey, 2011). The expression of genes in cancer cells was obtained by mapping RNA-sequence reads to the human reference genome or mouse reference genome. We subjected ‘protein_coding’ genes to spatial transcriptome analysis.

### Clustering and UMAP visualization

Data mining analyses such as clustering, UMAP analysis and DEG extraction were performed with the functions ‘runPCA’, ‘FindNeighbors’, ‘FindClusters’, ‘runUMAP’ and ‘FindAllMarkers’ in Seurat version 3.2. (Stuart et al., 2019). Cell cycle estimation was performed by the function ‘CellCycleScoring’ using cell cycle marker genes in Seurat. A heatmap of DEGs (adjusted *P*-value<0.05 and pct.1−pct.2>0.1) (Tables S2 and S4) was drawn using ComplexHeatmap (Gu et al., 2016). These packages and functions were run in R version 3.6.3.

### Enrichment analysis using DEGs

Pathway and GO enrichment analyses were performed by Metascape (https://metascape.org/gp/index.html#/main/step1) (Zhou et al., 2019). DEGs from each cluster were subjected to the Metascape interface. Differential enrichment terms were analyzed in multiple gene list mode. The results of the enrichment analysis were visualized as heatmaps.

### Immunostaining of tumor sections

Primary tumors generated from transplanted MDA-MB-231 cells were fixed in 4% paraformaldehyde overnight and embedded in paraffin. Primary tumor sections were dewaxed with xylene and rehydrated with ethanol (100% to 70%). Antigen retrieval was performed by boiling the specimens in Immunosaver (Nissin EM, Tokyo, Japan) diluted 1:200 for 45 min at 98°C. The sections were permeabilized with 0.1% Triton X-100 (Sigma-Aldrich, St Louis, MO, USA) for 15 min. After blocking with Dako blocking reagent (Vector Laboratories, Newark, CA, USA) for 30 min, sections were incubated with primary antibodies for 1 h at room temperature. Sections were then incubated with the primary antibodies, anti-CD44 (60224-1-IG, Proteintech Group, Rosemont, IL, USA; diluted 1:100) and anti-HMGA1 (ab252930, Abcam, Cambridge, UK; diluted 1:200), and secondary anti-mouse Alexa Fluor 488-labeled antibody (A-11001, Thermo Fisher Scientific, Waltham, MA, USA; diluted 1:1000) and anti-rabbit Alexa Fluor 594-labeled antibody (A-212-7, Thermo Fisher Scientific; diluted 1:1000). Slides were mounted with VECTASHIELD mounting medium with Hoechst 33342 (Thermo Fisher Scientific). Stained sections were imaged using an FV10i Laser Scanning Microscope (OLYMPUS, Tokyo, Japan).

### Analysis of the public scRNA-seq dataset

The scRNA-seq dataset of the MDA-MB-231-LM2 xenograft model (GSE163210) (Moravec et al., 2021 preprint) was downloaded from the GEO database. The scRNA-seq datasets of TNBC patients from the public datasets GSE161529 (Pal et al., 2021), GSE176078 (Wu et al., 2021b) and GSE180286 (Xu et al., 2021) were also downloaded from the GEO database. The scRNA-seq datasets were analyzed with Seurat vignettes, and the integration of datasets was performed by Harmony (Korsunsky et al., 2019). Low-quality single cells (nFeature_RNA<500 and percent.mt>20) were removed. The clinical datasets were analyzed with clinical annotation data.

### Statistical analysis

Chi-squared test, Fisher's exact test and log rank test were performed in R. DEG extraction was performed by the ‘FindAllMarkers’ function with the Wilcoxon rank sum test. Significance was defined as *P*<0.05.

## Supplementary Material

Supplementary information
