# Supplementary material for: Identification of two cancer stem cell-like populations in triple-negative breast cancer xenografts
Source: Dis Model Mech. 2022 Jun 23;15(6):dmm049538. doi: 10.1242/dmm.049538 (PMC9235877; doi:10.1242/dmm.049538)
Supplement: Supplementary information [file dmm-15-049538-s1.pdf]

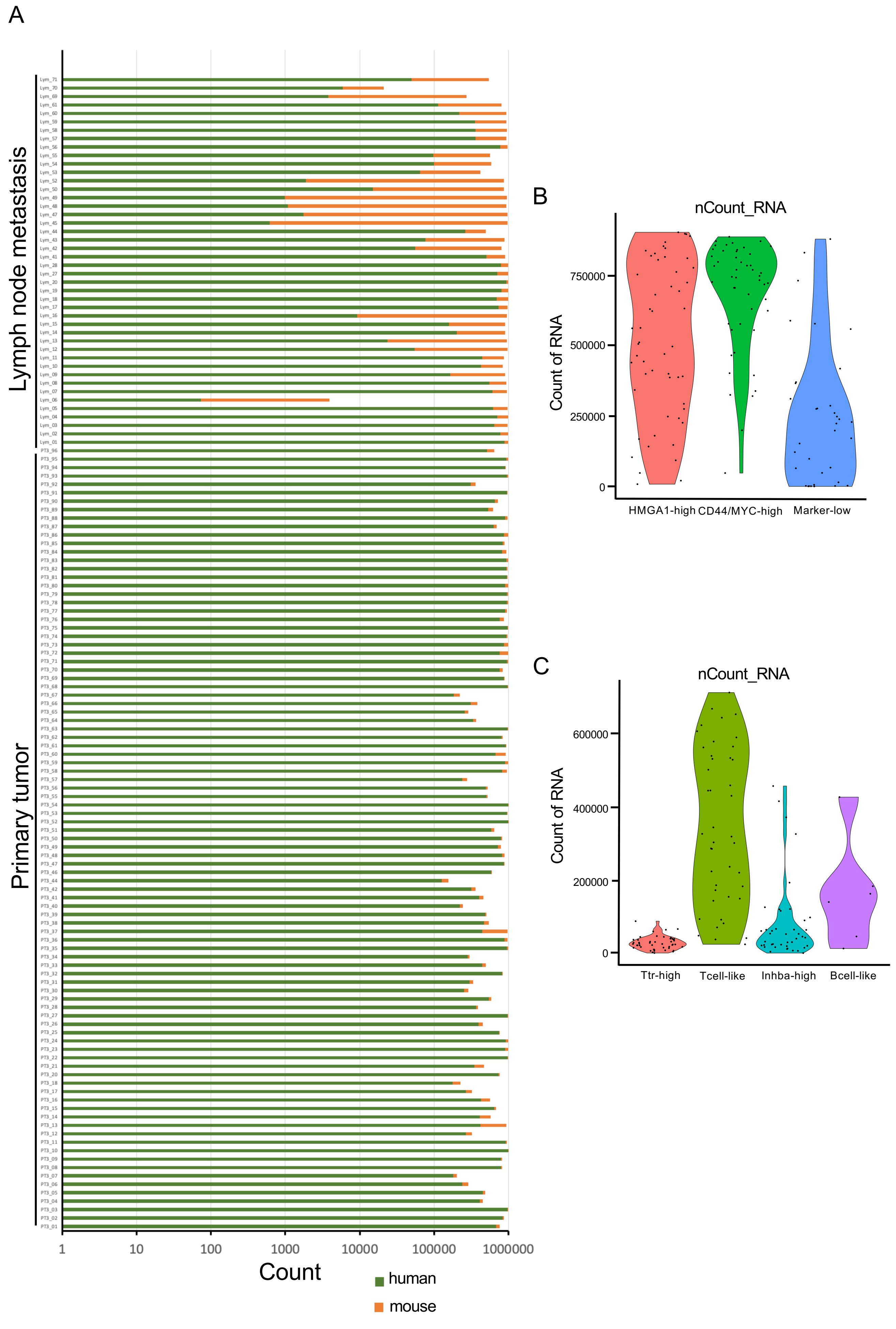

Fig. S1. Read counts and heterogeneity in each spot. (A) Read counts of human and mouse transcripts in each spot. (B) Violin plot of read counts in human cancer clusters. (C) Violin plot of read counts in mouse stromal clusters.

Disease Models & Mechanisms • Supplementary information

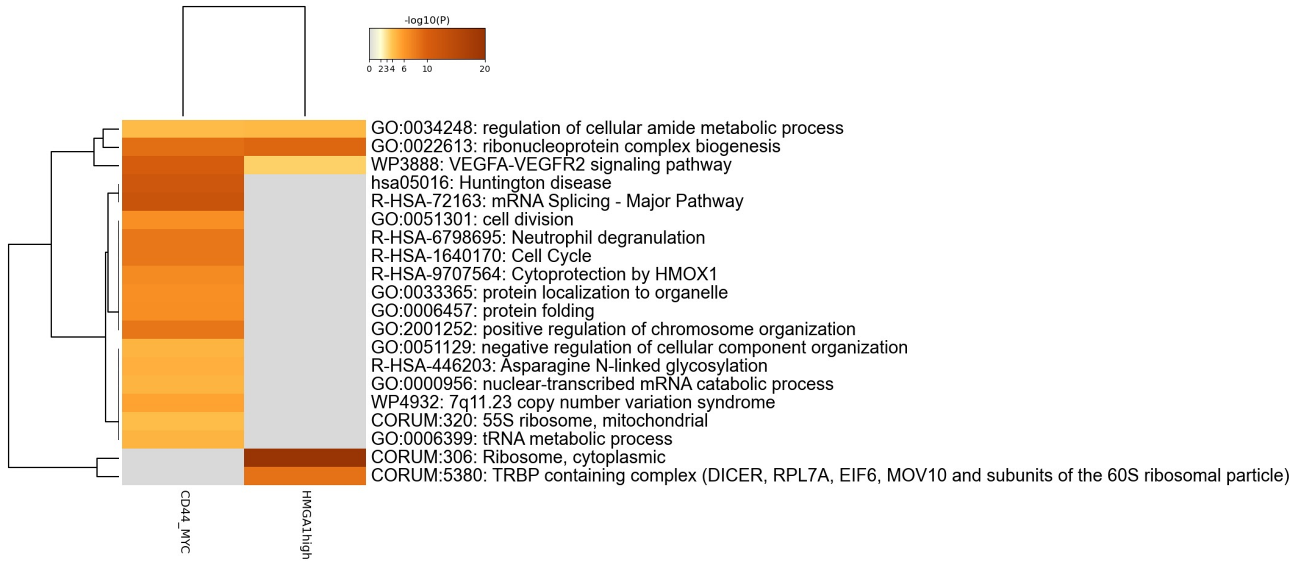

Fig. S2. Enrichment analysis of DEGs in the two CSC-like populations. Heatmap of the results of the enrichment analysis of the differentially expressed genes (DEGs) in the HMGA1-high and CD44/MYC-high clusters using Metascape.

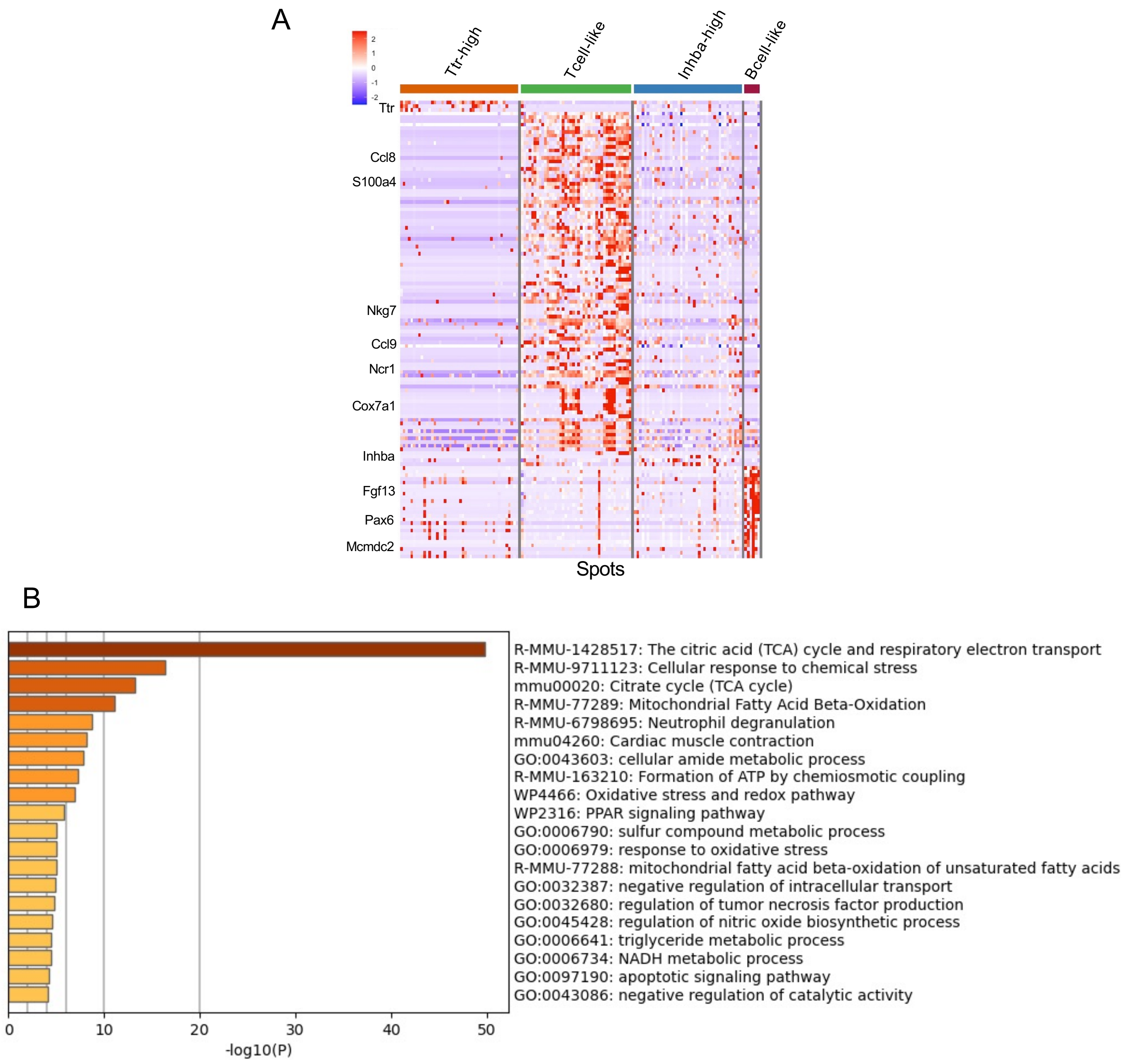

Fig. S3. Stromal cluster DEG analysis and enrichment analysis. (A) Heatmap of the differentially expressed genes (DEGs) (adjusted p value < 0.05 and pct.1 - pct.2 > 0.1) in mouse stromal cells. (B) Heatmap of the results of the enrichment analysis in the Tcell-like cluster.

Disease Models & Mechanisms • Supplementary information

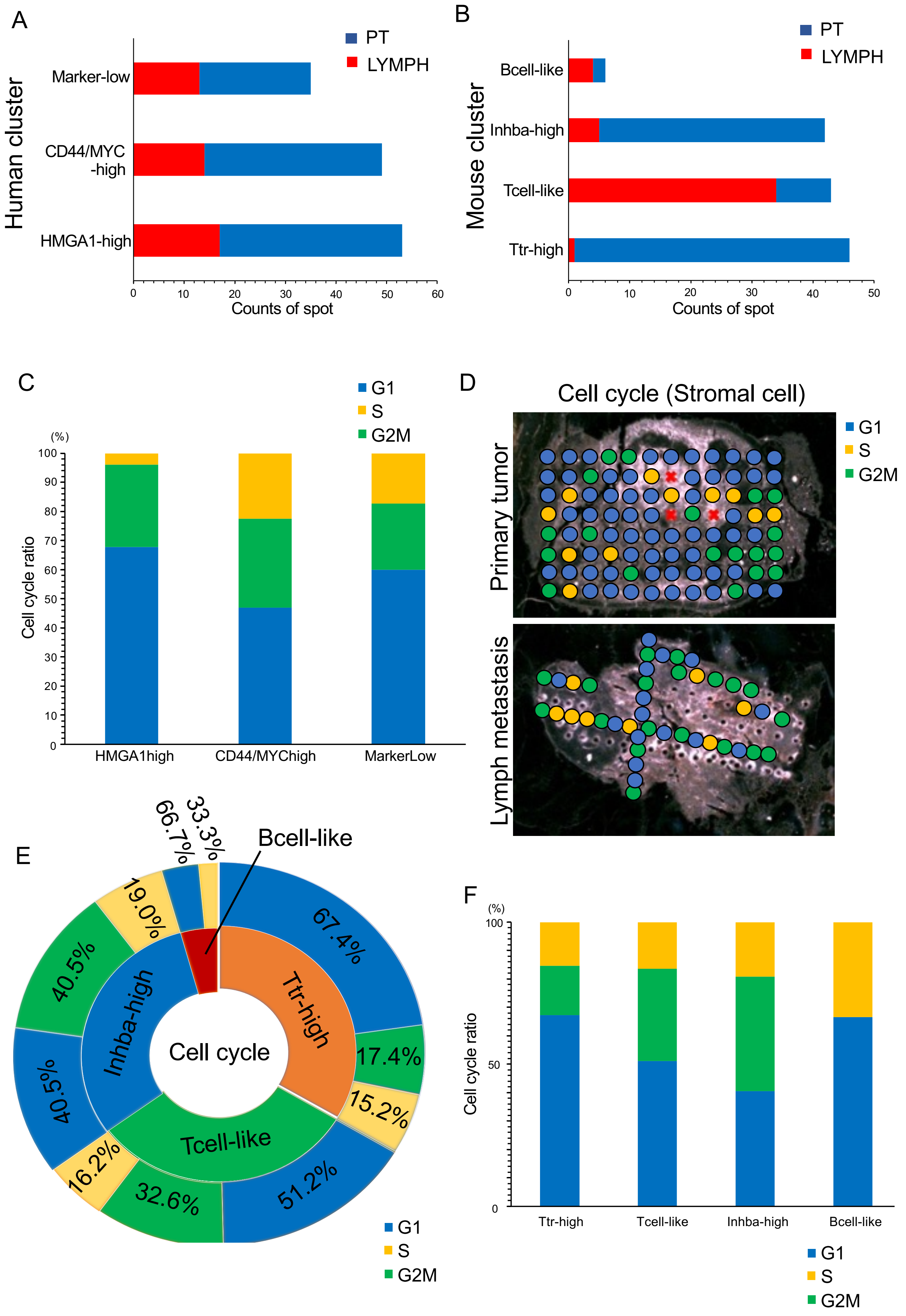

Fig. S4. Spatial analysis of mouse clusters in the primary tumor and lymph node metastasis. (A,B) Bar plots of spot counts in the primary tumor and lymph node metastasis. (A) human and (B) mouse. (C) Cell cycle phase proportions of the human cancer clusters. (D) Cell cycle proportions of the mouse stromal cells in the primary tumor and lymph node metastasis. (E) Sunburst plot of the cell cycle in mouse stromal cell clusters. (F) Cell cycle proportions of the mouse stromal clusters.

Disease Models & Mechanisms • Supplementary information

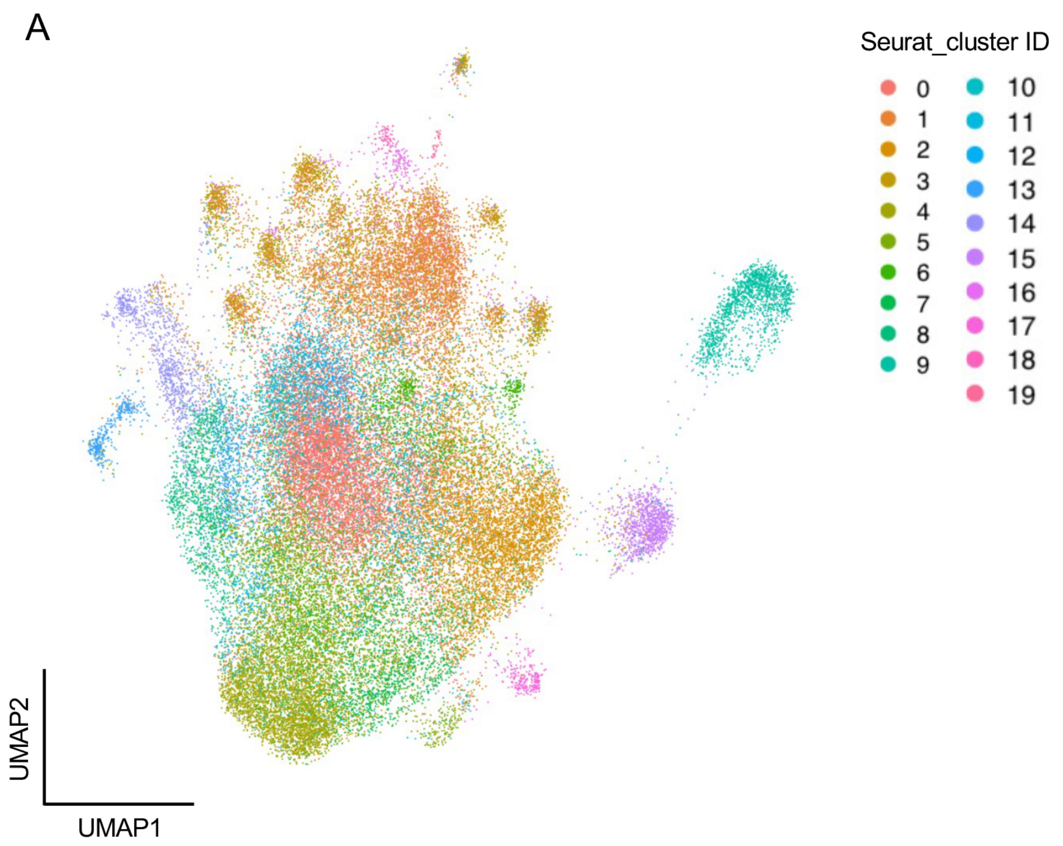

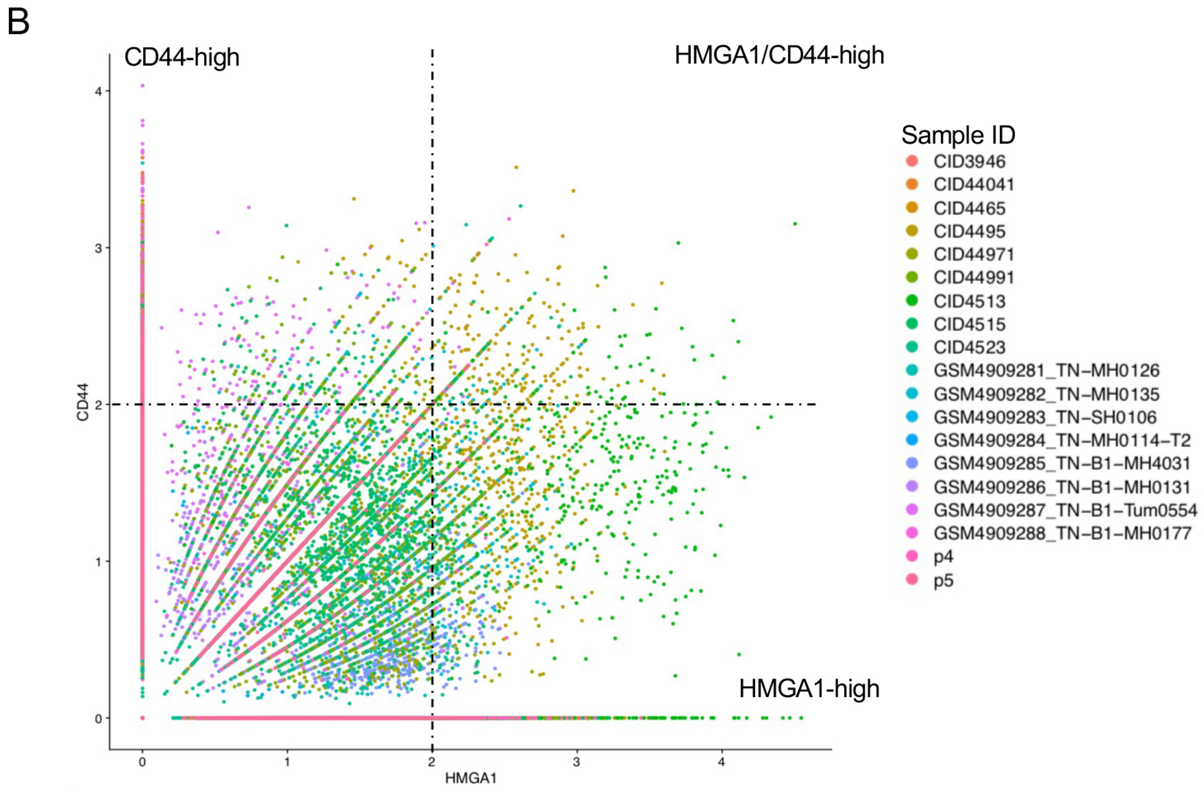

Fig. S5. Reanalysis of integrated scRNA-seq data. (A) UMAP plot of cancer cells with cluster identification by Seurat. (B) Scatter plot of CD44 and HMGA1 expression in the integrated datasets.

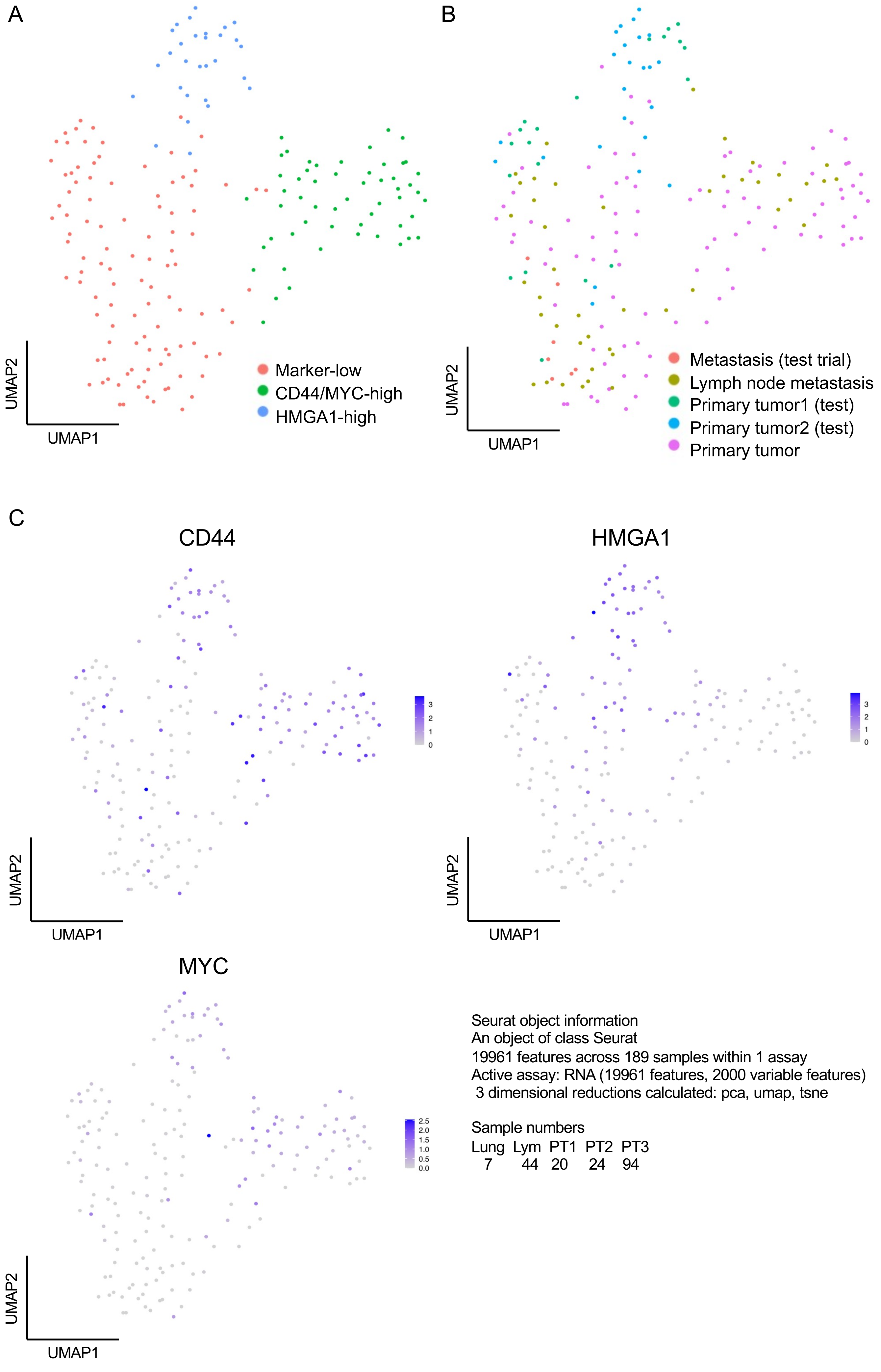

Fig. S6. Integrated analysis of spatial transcriptomics including other xenografting tumors. (A) UMAP plot of cancer cells with cluster identification by Seurat. (B) UMAP plot cancer cells with xenografting sample identification. (C) Expression analysis of CD44, HMGA1, and MYC in human cancer clusters including pilot samples.

Table S1. Clustering of micro-spots

Click here to download Table S1

Table S2. Marker genes in human cancer cells

Click here to download Table S2

Table S3. Upstream enrichment analysis of cancer cell clusters

Click here to download Table S3

Table S4. GO enrichment analysis of cancer cell clusters

Click here to download Table S4

Table S5. Marker genes in mouse stromal cells

Click here to download Table S5

Table S6. Cell cycle index in clusters

Click here to download Table S6

Table S7. Cell annotation in TNBC patients

Click here to download Table S7

Table S8. DEGs between CSC-like populations in TNBC patients and overlapped genes

Click here to download Table S8
